# Supplementary material for: Intolerance-of-uncertainty therapy versus metacognitive therapy for generalized anxiety disorder in primary health care: A randomized controlled pilot trial
Source: PLoS One. 2023 Jun 14;18(6):e0287171. doi: 10.1371/journal.pone.0287171 (PMC10266649; doi:10.1371/journal.pone.0287171)
Supplement: S1 Checklist — (DOC) [file pone.0287171.s001.doc]

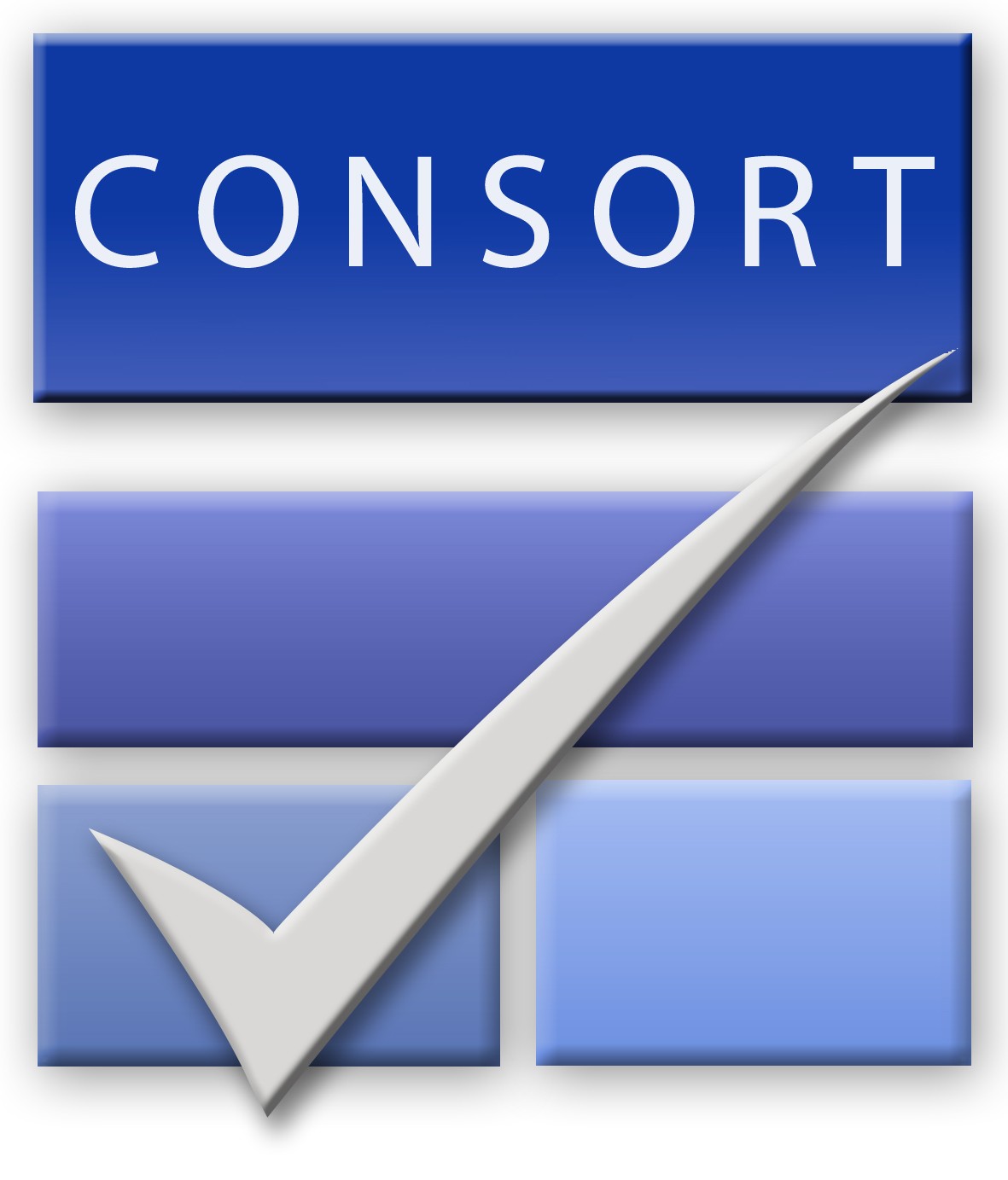
CONSORT 2010 checklist of information to include when reporting a pilot or feasibility trial*

| Section/Topic | Item No | Checklist item GAD-studien | Reported on page No |
| --- | --- | --- | --- |
| Title and abstract | | | |
|  | 1a | Identification as a pilot or feasibility randomised trial in the title | Yes p 1 |
|  | 1b | Structured summary of pilot trial design, methods, results, and conclusions (for specific guidance see CONSORT abstract extension for pilot trials) | Yes p 2 |
| Introduction | | | |
| Background and objectives | 2a | Scientific background and explanation of rationale for future definitive trial, and reasons for randomised pilot trial | Yes pp. 3-6 |
|  | 2b | Specific objectives or research questions for pilot trial | Yes p 6 |
| Methods | | | |
| Trial design | 3a | Description of pilot trial design (such as parallel, factorial) including allocation ratio | Yes pp. 6-13 |
|  | 3b | Important changes to methods after pilot trial commencement (such as eligibility criteria), with reasons | Not appl |
| Participants | 4a | Eligibility criteria for participants | Yes, p 7 |
|  | 4b | Settings and locations where the data were collected | Yes p 7 |
|  | 4c | How participants were identified and consented | Yes pp. 7-14 |
| Interventions | 5 | The interventions for each group with sufficient details to allow replication, including how and when they were actually administered | Yes, pp. 7-9 |
| Outcomes | 6a | Completely defined prespecified assessments or measurements to address each pilot trial objective specified in 2b, including how and when they were assessed | Yes pp.9-12 |
|  | 6b | Any changes to pilot trial assessments or measurements after the pilot trial commenced, with reasons | Not appl |
|  | 6c | If applicable, prespecified criteria used to judge whether, or how, to proceed with future definitive trial | Yes pp.26-28 |
| Sample size | 7a | Rationale for numbers in the pilot trial | Yes p 6 |
|  | 7b | When applicable, explanation of any interim analyses and stopping guidelines | Not appl |
| Randomisation: |  |  |  |
| Sequence  generation | 8a | Method used to generate the random allocation sequence | Yes pp.12-13 |
|  | 8b | Type of randomisation(s); details of any restriction (such as blocking and block size) | Yes pp. 12-13 |
| Allocation  concealment  mechanism | 9 | Mechanism used to implement the random allocation sequence (such as sequentially numbered containers), describing any steps taken to conceal the sequence until interventions were assigned | Yes pp.12-13 |
| Implementation | 10 | Who generated the random allocation sequence, who enrolled participants, and who assigned participants to interventions | Yes pp.12-13 |
| Blinding | 11a | If done, who was blinded after assignment to interventions (for example, participants, care providers, those assessing outcomes) and how | Not appl |
|  | 11b | If relevant, description of the similarity of interventions | Yes pp.4-6, 24-29 |
| Statistical methods | 12 | Methods used to address each pilot trial objective whether qualitative or quantitative | Yes pp.13-15 |
| Results | | | |
| Participant flow (a diagram is strongly recommended) | 13a | For each group, the numbers of participants who were approached and/or assessed for eligibility, randomly assigned, received intended treatment, and were assessed for each objective | Yes pp.12-13, fig 1, 16-17 |
|  | 13b | For each group, losses and exclusions after randomisation, together with reasons | Yes p13 fig 1, pp.16-17 |
| Recruitment | 14a | Dates defining the periods of recruitment and follow-up | Yes p16 |
|  | 14b | Why the pilot trial ended or was stopped | Yes p16 |
| Baseline data | 15 | A table showing baseline demographic and clinical characteristics for each group | Yes p16 |
| Numbers analysed | 16 | For each objective, number of participants (denominator) included in each analysis. If relevant, these numbers  should be by randomised group | Yes p13 fig 1, pp.16-20 |
| Outcomes and estimation | 17 | For each objective, results including expressions of uncertainty (such as 95% confidence interval) for any  estimates. If relevant, these results should be by randomised group | Yes pp.16-24 |
| Ancillary analyses | 18 | Results of any other analyses performed that could be used to inform the future definitive trial | Yes pp.16-24 |
| Harms | 19 | All important harms or unintended effects in each group (for specific guidance see CONSORT for harms) | Yes pp.16-24 |
|  | 19a | If relevant, other important unintended consequences | Yes pp.16-24 |
| Discussion | | | |
| Limitations | 20 | Pilot trial limitations, addressing sources of potential bias and remaining uncertainty about feasibility | Yes pp.27-29 |
| Generalisability | 21 | Generalisability (applicability) of pilot trial methods and findings to future definitive trial and other studies | Yes pp.24-29 |
| Interpretation | 22 | Interpretation consistent with pilot trial objectives and findings, balancing potential benefits and harms, and  considering other relevant evidence | Yes pp. 24-29 |
|  | 22a | Implications for progression from pilot to future definitive trial, including any proposed amendments | Yes pp. 24-29 |
| Other information | | |  |
| Registration | 23 | Registration number for pilot trial and name of trial registry | Yes pp.1,33 |
| Protocol | 24 | Where the pilot trial protocol can be accessed, if available | Yes p 33 |
| Funding | 25 | Sources of funding and other support (such as supply of drugs), role of funders | Yes p 33 |
|  | 26 | Ethical approval or approval by research review committee, confirmed with reference number | Yes pp.15-16, 33 |

Citation: Eldridge SM, Chan CL, Campbell MJ, Bond CM, Hopewell S, Thabane L, et al. CONSORT 2010 statement: extension to randomised pilot and feasibility trials. BMJ. 2016;355.

*We strongly recommend reading this statement in conjunction with the CONSORT 2010, extension to randomised pilot and feasibility trials, Explanation and Elaboration for important clarifications on all the items. If relevant, we also recommend reading CONSORT extensions for cluster randomised trials, non-inferiority and equivalence trials, non-pharmacological treatments, herbal interventions, and pragmatic trials. Additional extensions are forthcoming: for those and for up to date references relevant to this checklist, see [www.consort-statement.org](http://www.consort-statement.org/).
