# Supplementary material for: Intolerance-of-uncertainty therapy versus metacognitive therapy for generalized anxiety disorder in primary health care: A randomized controlled pilot trial
Source: PLoS One. 2023 Jun 14;18(6):e0287171. doi: 10.1371/journal.pone.0287171 (PMC10266649; doi:10.1371/journal.pone.0287171)
Supplement: S1 File — (DOCX) [file pone.0287171.s002.docx]

**Study protocol translated from Swedish**

**Comparison of the efficacy of two variants of cognitive behavioral therapy for generalized anxiety disorder in primary care: a randomized controlled pilot study**

**Background**

Mental illness is increasing in the population and constitutes significant suffering for the individual as well as high costs for healthcare and society in general (Socialstyrelsen, 2017). Generalized anxiety disorder (GAD) is a commonly occurring condition in primary care and estimates show that up to 25% of primary care patients seeking mental illness meet the criteria (Dugas & Robichaud, 2006). GAD is characterized by excessive and uncontrollable worry about a number of different events or activities for at least six months (American Psychiatric Association, 2013). The condition is often associated with increased irritability, restlessness, sleep disturbance, concentration difficulties and muscular tension with secondary pain. The condition has a chronic course and often leads to long-term suffering and functional impairment. Unfortunately, diagnostics and treatment are not optimal today. Research shows that GAD is underdiagnosed in primary care, one reason may be that secondary, diffuse bodily symptoms are often the primary reason the patient seeks care. This leads to unnecessary somatic investigations and treatment attempts. Patients with GAD are often high consumers of healthcare and in many cases have been extensively somatically investigated for their physical complaints, which entails great suffering for the individual and high costs for society (Roy-Byrne & Wagner, 2004; Wittchen, 2002)

Cognitive behavioral therapy (CBT) is the psychological treatment that has been shown to be most effective in GAD. A variant of CBT that has been developed specifically for GAD is intolerance of uncertainty therapy (IUT) (Dugas & Robichaud, 2006). Research has shown that IUT is an effective treatment method and it is today the most used psychological treatment for GAD. Central to IUT is that patients with GAD have difficulty coping with situations characterized by uncertainty and that one way of dealing with this is to worry. Patients often have the idea that worry serves several adaptive functions, such as helping them to solve problems and to prepare for negative situations. In IUT, the treatment focuses on training skills in coping with uncertainty through exposure and reconsidering positive beliefs about anxiety through so-called behavioral experiments (e.g. worry as problem solving) (Dugas & Robichaud, 2006).

Metacognitive therapy (MCT) is another variant of CBT and is based on theories of metacognition (Wells, 2008). It has been shown to be effective in several psychiatric disorders, i.a. GAD. As a form of psychotherapy, it has been established since the 1990s. The theoretical model for MCT has good research support and is today an evidence-based treatment for GAD (Normann, et al., 2014; Sadeghi, et al., 2015). MCT targets a particular form of thinking that occurs in most types of mental disorders/psychiatric problems. This form of thinking – cognitive attentional syndrome (CAS) – is characterized by worrying/ruminating, brooding, threat-focused attention and unhelpful behavioral strategies. CAS means that thoughts and feelings cannot be regulated in an optimal and flexible way, that psychological problems are perpetuated and that vulnerability to psychological problems remains. The treatment focus in MCT is to systematically reduce CAS activity and to practice better functioning emotional regulation and a more flexible thinking style. The method is structured and, according to the manual, feasible in 10 sessions. MCT is evaluated in several studies internationally and there are also two meta-analyses of MCT studies (Normann, et al., 2014; Sadeghi, et al., 2015). The results from both of these suggest that MCT is an effective psychological treatment method for depression and anxiety states and has a better effect both at the end of treatment and at follow-up 12 months later when compared to waitlist controls and other forms of CBT. However, the number of studies is few and they are done on small patient groups (Normann, et al., 2014). There are no studies conducted in Sweden. Internationally, there is only one study that compared IUT and MCT (van der Heiden, et al., 2012). The study was carried out in psychiatric outpatient care and the results were beneficial for MCT. The need for more research that compares these treatments for a condition as common and at the same time disabling in primary care as GAD is therefore great.

**Aims and research questions**

The aim of the study is to investigate in a pilot format the conditions for conducting a larger randomized and controlled study comparing the effect of MCT and IUT in GAD in primary care. In the pilot study, the feasibility is tested by investigating the recruitment possibilities of patients, the implementation of the measurement procedure, drop-outs from the treatments and the patients' adherence to treatment (completed sessions and homework assignments). Information about the patients' experience of undergoing the treatments is also collected through a questionnaire. In addition to this, a preliminary evaluation of the effect of MCT and IUT is carried out with statistical significance testing and calculation of effect size and the proportion of patients who are clinically significantly improved. The purpose of the study is to learn lessons about patients with GAD in primary care and thereby optimize the conditions for a full-scale randomized and controlled study.

Questions:

1. What does the recruitment base look like among patients with GAD in primary care and is it large enough for a full-scale randomized controlled trial?

2. To what extent do patients answer instruments for measuring symptoms, function and quality of life?

3. To what extent do patients complete the treatments and how large is the dropout rate?

4. What are the patients' experiences of undergoing these psychological treatments in a primary care setting?

5. What is the effect of the two treatments and is it different between them?

**Methods**

Liljeholmen's care center has roughly 28,000 listed patients and an extensive psychotherapy operation with both counselors and psychologists in a psychosocial team. All therapists in the team have at least basic psychotherapy training in CBT. In line with the mission of primary care to constitute first-line psychiatry, the team treats mild to moderate states of anxiety and depression.

Research subjects are recruited from patients who are 18 years of age and older and who seek a doctor at the health center for symptoms of mental illness or with unexplained somatic symptoms where investigation does not lead to a diagnosis. Patients who the doctor at the health center assesses that mental illness is the primary problem are referred to the psychosocial team for assessment, diagnostics and treatment. Practitioners conduct clinical interviews and structured diagnostics using the Mini-International Neuropsychiatric Interview (MINI; Sheehan et al., 1998). MINI is a validated, structured interview for diagnostics and differential diagnosis of mainly anxiety and depression conditions (SBU, 2012).

Patients who are judged to have GAD as a primary diagnosis and checked against inclusion and exclusion criteria are given information about the study and offered participation in it.

**Inclusion criteria**

1. Age at least 18 years.

2. Master the Swedish language without interpreter support.

**Exclusion criteria**

1. Ongoing use of narcotics or non-prescription prescription drugs (at least one month clean samples and six weeks for cannabis).

2. More severe psychiatric disorder, such as bipolar disorder or psychosis, more severe depression, suicidality and diagnosed cognitive impairment.

3. Treatment with psychotropic drugs that is initiated or changed less than six weeks before treatment or during treatment. Ongoing stable medication during treatment is accepted.

4. Other concurrent psychological treatment.

Patients who wish to participate in the study receive oral and written information and give their written consent to it. They then have to answer a battery of instruments (self-assessment questionnaires) and are then randomized to treatment with MCT or IUT. Randomization is done by a person independent of the project. A research nurse collects the patients' responses to instruments, notifies them of the treatment they have been allocated to and books them in with therapists. Both treatments are manual-based with a specified session content and can include up to 12 sessions. The treatment is carried out individually. A different therapist than the one who made the diagnostic assessment treats the patient. If the patient is no longer judged to meet the criteria for excessive and uncontrollable anxiety, treatment can be terminated before the twelfth session. Audio recordings are made of therapy sessions during the pilot study with patients' consent solely to assess therapist competence and adherence to manuals.

**Treatment arms:**

1. IUT according to Dugas and Robichaud's treatment manual (2006), up to 12 sessions

2. MCT according to Wells' treatment manual (2008), up to 12 sessions

**Survey variables**

Therapists participating in the study answer a questionnaire with questions about education, clinical experience, etc. (Appendix 5 a). Patients participating in the study answer a questionnaire with questions about education, how long they have had problems with anxiety, previously completed psychological treatment, etc. (Appendix 5 b). Psychiatric diagnosis in patients is assessed by therapists using the MINI. Competence in MCT is measured with the Metacognitive Therapy Competency Scale (Nordahl & Wells, 2009), competence in IUT is measured with the Cognitive Therapy Scale-Revised (Blackburn, et al., 2001). Adherence to treatment is measured with a checklist for session content (see example in Appendix 12). Patients' experiences of having participated in the treatment are measured with an evaluation form (Appendix 13). The impact evaluation takes place with the following four self-assessment instruments:

1) pathological worry measured with the Penn State Worry Questionnaire (PSWQ; Meyer, et al., 1990),

2) depressive symptoms with the Patient Health Questionnaire 9 (PHQ-9; Kroenke, et al., 2001),

3) functional level with WHODAS 2.0 (National Board of Health and Welfare, 2015) and

4) life satisfaction/quality of life with the Satisfaction with Life Scale (SWLS; Diener et al., 1985).

The total number of questions in the four instruments is 46, which is estimated to take approx. 15 minutes to complete.

Administration time for the instruments:

| Instrument | Number of questions | Pre treatment | Mid treatment after session 6 | Post treatment | Follow-up after 6 mounths |
| --- | --- | --- | --- | --- | --- |
| PSWQ | 16 | X | X | X | X |
| PHQ-9 | 10 | X | X | X | X |
| WHODAS 2.0 | 15 | X |  | X | X |
| SWLS | 5 | X |  | X | X |
| Number of questions | 46 | 46 | 26 | 46 | 46 |

**Time schedule**

A total of 50 patients are planned to be included in the pilot study. Data collection is expected to take place between April 2018 and October 2019.

**Data processing**

Results for questions 1-4 are based on data from the self-assessment instruments completed by the patients, the background questionnaires, the evaluation questionnaire, medical record data regarding adherence to treatment and the therapist's diagnostic assessment and will be described descriptively. Likewise, the data from the background questionnaire of the therapists is presented descriptively in order to be able to describe the two groups of therapists. The audio recordings made of therapy sessions during the pilot study are used solely to assess therapist competence and adherence to manuals

Based on existing data and based on the conditions, we plan to make a preliminary evaluation of the effect of MCT and IUT with statistical significance testing and calculation of effect size and the proportion of patients who are clinically significantly improved.

**Previous studies and experience**

Both IUT and MCT are validated and potent psychological treatment methods for GAD that are both used in clinical practice and have been used by several of the researchers in the research group. Several of the researchers in the research group already have many years of experience with studies on CBT for patients with depression and anxiety disorders in psychiatric outpatient care, including recruitment, measurement and treatment.

**Access to relevant security/personnel**

The pilot study is carried out at the Liljeholmen health center within the framework of everyday clinical work and the patients are recruited from among the health center's patients. For the patients, the care will not differ from what they would have received outside the study, with the exception that they will be asked to complete the self-assessment instruments to a greater extent than is usually the case and that the treatment sessions will be recorded. The therapists are employed staff at Liljeholmen's health center as well as clinically active psychologists employed at the Competence Center for Psychotherapy. The therapists initially receive regular guidance in each method from experienced clinicians and supervisors. A research nurse will be engaged on a part-time basis to follow the patients through the study, ensure that they answer the self-assessment instruments and book appointments with the therapists.

**Ethical considerations**

Receiving an anxiety diagnosis can be experienced as stigmatizing, especially for those patients who previously had no knowledge that there could be a psychological explanation for somatic complaints. However, a possible negative initial reaction is outweighed by the possibility of a correct diagnosis and thus access to adequate treatment. Patients are randomly allocated to different treatments and do not know in advance which type of treatment they will receive. This limits their self-determination, which is otherwise indicative in clinical treatment. However, the study's questions require this design. In addition, the two treatments are both variants of CBT and previous research has shown them to be potent in the treatment of anxiety problems. The project aims to investigate the feasibility of recruiting, measuring and treating patients with GAD in primary care, knowledge that is planned to form the basis for a full-scale randomized controlled trial whose purpose is to investigate which treatment (MCT or IUT) is most effective. Patients' participation in the study is voluntary and they can cancel at any time without changing their opportunities for good care. Patients who refuse to participate or cancel their participation are offered usual treatment. During assessment and treatment, patients are asked to report any unwanted events that occur within the scope of the study to Liljeholmen's medical center's operations manager for documentation and action. We assess the risk of unauthorized persons accessing patient data as virtually non-existent in the project. Consent forms are kept in locked areas at Liljeholmen's health center and only the operations manager, authorized researchers and the research nurse have access to them. Other patient data that is collected is de-identified and only the operations manager, principal researcher and research nurse have access to the code key.

**Meaning**

Mental illness is common but not always noticed among primary care patients and GAD in particular is often underdiagnosed. It is therefore important to improve identification and handling of the condition. A systematic diagnosis of GAD in primary care can provide an earlier and correct diagnosis, which is the prerequisite for adequate treatment. There is generally a lack of studies of psychological treatment of mental illness in primary care and this also applies to patients with GAD. Although CBT is the psychological treatment that is most effective in GAD, there is a lack of knowledge about which variant of CBT is the more effective. The project is expected to provide knowledge about improved identification and handling of patients with GAD in primary care, and contribute to the knowledge base about the effectiveness of different variants of CBT. If MCT proves to be at least as effective as IUT, it means that the arsenal of psychological treatments for GAD in primary care should be broadened. As the study is conducted in regular primary care, the results of the study are expected to be generalizable to primary care as a whole, provided they are replicated in a more full-scale randomized controlled trial.

**References**

**American Psychiatric Association**.(2013). MINI-D IV. Diagnostiska kriterier enligt DSM-IV. Pilgrim Press: Danderyd.

**Blackburn**, I.-M., James, I. A., Milne, D. L., Baker, C., Standart, S., Garland, A. & Reichelt, F. K. (2001). The Revised Cognitive Therapy Scale (CTS-R): Psychometric properties. *Behavioural and Cognitive Psychotherapy, 29*, 431–446.

**Diener**, E., Emmons, R. A., Larsen, R. J., & Griffin, S. (1985). The satisfaction with life scale. *Journal of Personality Assessment, 49*(1), 71–75.

**Dugas M**. ,& Robichaud, M. (2006) *Cognitive-Behavioral Treatment for Generalized Anxiety Disorder, From Science to Practice*. Routledge

**Kroenke**, K., Spitzer, R. L., & Williams, J. B. (2001). The PHQ-9: validity of a brief depression severity measure. *Journal of General Internal Medicine, 16*(9), 606-613.

**Meyer**, T. J., Miller, M. L., Metzger, R. L., & Borkovec, T. D. (1990). Development and validation of the Penn State Worry Questionnaire. *Behaviour Research and Therapy, 28*(6), 487-495.

**Nordahl**, H. M., & Wells, A. (2009). *Metacognitive Therapy Competency Scale*. Metacognitive Therapy Institute.

**Normann, N**., van Emmerik, A. A., & Morina, N. (2014). The efficacy of metacognitive therapy for anxiety and depression: a meta-analytic review. [Meta-Analysis]. *Depression and anxiety, 31*(5), 402-411.

**Roy-Byrne**, P. P., & Wagner, A. (2004). Primary care perspectives on generalized anxiety disorder. [Review]. *The Journal of clinical psychiatry, 65 Suppl 13*, 20-26.

**Sadeghi,** R., Mokhber, N., Mahmoudi, L. Z., Asgharipour, N., & Seyfi, H. (2015). A systematic review and meta-analysis on controlled treatment trials of metacognitive therapy for anxiety disorders. [Review]. *Journal of research in medical sciences : the official journal of Isfahan University of Medical Sciences, 20*(9), 901-909.

**SBU**. Diagnostik och uppföljning av förstämningssyndrom. En systematisk litteraturöversikt. Stockholm: Statens beredning för medicinsk utvärdering (SBU); 2012. SBU-rapport nr 212. ISBN 978-91-85413-52-2.

**Sheehan**, D. V., Lecrubier, Y., Sheehan, K. H., Amorim, P., Janavs, J., Weiller, E., et al. (1998). The Mini-International Neuropsychiatric Interview (M.I.N.I.): the development and validation of a structured diagnostic psychiatric interview for DSM-IV and ICD-10. [Review]. *The Journal of clinical psychiatry, 59 Suppl 20*, 22-33;quiz 34-57.

**Socialstyrelsen.** Mätning av hälsa och funktionshinder 2015. Manual till WHO:s formulär för bedömning av funktionshinder, WHO Disability Assessment Schedule. WHODAS 2.0 Artikelnr 2015-5-1.

**Socialstyrelsen.** Nationella riktlinjer för vård vid depressions- och ångestsyndrom 2017 − stöd för styrning och ledning. Stockholm: Socialstyrelsen; 2017. Artikelnr 2017-12-4.

**van der Heiden**, C., Muris, P., & van der Molen, H. T. (2012). Randomized controlled trial on the effectiveness of metacognitive therapy and intolerance-for-uncertainty therapy for generalized anxiety disorder. *Behavior Research and Therapy, 50*, 100-109.

**Wells**, A. (2008). *Metacognitive Therapy for Anxiety and Depression*. New York: Guilford Press.

**Wittchen**, H. U. (2002). Generalized anxiety disorder: prevalence, burden, and cost to society. *Depression and anxiety, 16*(4), 162-171.
