# Supplementary material for: Intolerance-of-uncertainty therapy versus metacognitive therapy for generalized anxiety disorder in primary health care: A randomized controlled pilot trial
Source: PLoS One. 2023 Jun 14;18(6):e0287171. doi: 10.1371/journal.pone.0287171 (PMC10266649; doi:10.1371/journal.pone.0287171)
Supplement: S2 File — (DOCX) [file pone.0287171.s003.docx]

**Jämförelse av effekten mellan två varianter av kognitiv beteendeterapi vid generaliserat ångestsyndrom i primärvården: en randomiserad kontrollerad pilotstudie**

**Bakgrund**

Psykisk ohälsa ökar i befolkningen och utgör ett betydande lidande för individen samt höga kostnader för sjukvården och samhället i övrigt (Socialstyrelsen, 2017). Generaliserat ångestsyndrom (generalized anxiety disorder, GAD) är ett vanligt förekommande tillstånd inom primärvården och beräkningar visar att upp till 25 % av primärvårdspatienter som söker för psykisk ohälsa uppfyller kriterierna (Dugas & Robichaud, 2006). GAD kännetecknas av överdriven och okontrollerbar oro inför ett antal olika händelser eller aktiviteter under minst sex månader (American Psychiatric Association, 2013). Tillståndet är ofta förknippat med ökad irritabilitet, rastlöshet, sömnstörning, koncentrationssvårigheter samt muskulär spänning med sekundär smärta. Tillståndet har ett kroniskt förlopp och leder ofta till långvarigt lidande och funktionsnedsättning. Tyvärr är dock inte diagnostik och behandling optimala idag. Forskning visar att GAD är underdiagnostiserat i primärvården, en anledning kan vara att sekundära, diffusa kroppsliga symtom ofta är den primära orsaken till att patienten söker vård. Detta leder till onödiga somatiska utredningar och behandlingsförsök. Patienter med GAD är ofta högkonsumenter av sjukvård och har i många fall blivit extensivt somatiskt utredda för sina kroppsliga besvär, vilket medför stort lidande för individen och höga kostnader för samhället (Roy-Byrne & Wagner, 2004; Wittchen, 2002).

Kognitiv beteendeterapi (KBT) är den psykologiska behandling som visats vara mest effektiv vid GAD. En variant av KBT som har utvecklats specifikt för GAD är intolerans för osäkerhet-terapi (intolerance of uncertainty therapy, IUT) (Dugas & Robichaud, 2006). Forskning har visat att IUT är en effektiv behandlingsmetod och den är idag den mest använda psykologiska behandlingen vid GAD. Centralt i IUT är att patienter med GAD har en svårighet att stå ut med situationer som präglas av osäkerhet och att ett sätt att hantera det är att oroa sig. Patienter har ofta föreställningen att oro fyller flera adaptiva funktioner som till exempel kan hjälpa dem att lösa problem och att förbereda sig inför negativa situationer. I IUT fokuserar behandlingen på att träna färdigheter i att stå ut med osäkerhet genom exponering och att ompröva positiva föreställningar om oro genom s.k. beteendeexperiment (t.ex. oro som problemlösning) (Dugas & Robichaud, 2006).

Metakognitiv terapi (metacognitive therapy, MCT) är en annan variant av KBT och baseras på teorier om metakognition (Wells, 2008). Den har visat sig vara effektiv vid flera psykiatriska störningar, bl.a. GAD. Som psykoterapiform har den varit etablerad sedan 1990-talet. Den teoretiska modellen för MCT har gott forskningsstöd och är idag en evidensbaserad behandling vid GAD (Normann, et al., 2014; Sadeghi, et al., 2015). MCT riktar sig mot en särskild form av tänkande som förekommer vid de flesta typer av psykiska besvär/psykiatrisk problematik. Denna form av tänkande – kognitivt uppmärksamhetssyndrom (cognitive attentional syndrome, CAS) – kännetecknas av oro/grubblande, ältande, hotfokuserad uppmärksamhet och ohjälpsamma beteendestrategier. CAS medför att tankar och känslor inte kan regleras på ett optimalt och flexibelt sätt, att psykiska problem vidmakthålls och att sårbarhet för psykiska besvär kvarstår. Behandlingsfokus i MCT är att på ett systematiskt sätt minska CAS-aktivitet och att öva upp en bättre fungerande känslomässig reglering och en mer flexibel tankestil. Metoden är strukturerad och enligt manual genomförbar på 10 sessioner. MCT är utvärderad i flertalet studier internationellt och det finns även två metaanalyser av MCT-studier (Normann, et al., 2014; Sadeghi, et al., 2015). Resultaten från båda dessa tyder på att MCT är en effektiv psykologisk behandlingsmetod vid depression och ångesttillstånd och har bättre effekt både vid avslutad behandling och uppföljning 12 månader senare när man jämför med väntelistekontroller och andra former av KBT. Antalet studier är dock få och de är gjorda på små patientgrupper (Normann, et al., 2014). Det finns inga studier genomförda i Sverige. Internationellt finns det endast en studie som jämfört IUT och MCT (van der Heiden, et al., 2012). Studien genomfördes inom psykiatrisk öppenvård och resultatet var till fördel för MCT. Behovet av mer forskning som jämför dessa behandlingar vid ett inom primärvården så pass vanligt förekommande och samtidigt funktionsnedsättande tillstånd som GAD är därför stort.

**Syfte och frågeställningar**

Syftet med studien är att i ett pilotformat undersöka förutsättningarna att genomföra en större randomiserad och kontrollerad studie som jämför effekten av MCT och IUT vid GAD inom primärvården. I pilotstudien testas genomförbarheten genom att undersöka rekryteringsmöjligheter av patienter, mätprocedurens genomförande, bortfall från behandlingarna samt patienternas följsamhet till behandling (genomförda sessioner och hemuppgifter). Information om patienternas upplevelse av att genomgå behandlingarna samlas också in genom en enkät. Utöver detta genomförs en preliminär utvärdering av effekten av MCT och IUT med statistisk signifikansprövning samt beräkning av effektstorlek och andelen patienter som är kliniskt signifikant förbättrade. Syftet med studien är att dra lärdomar om patienter med GAD i primärvården och därmed optimera förutsättningarna för en fullskalig randomiserad och kontrollerad studie.

Frågeställningar:

1. Hur ser rekryteringsunderlaget ut bland patienter med GAD i primärvård och är det tillräckligt stort för en fullskalig randomiserad kontrollerad studie?
2. I vilken utsträckning besvarar patienter instrument för mätning av symtom, funktion och livskvalitet?
3. I vilken grad fullföljer patienterna behandlingarna och hur stort är bortfallet?
4. Vad är patienternas upplevelser av att genomgå dessa psykologiska behandlingar i primärvårdsmiljö?
5. Vilken effekt har de två behandlingarna och skiljer den sig mellan dem?

**Metod**

Liljeholmens vårdcentral har drygt 28 000 listade patienter och en omfattande psykoterapiverksamhet med såväl kuratorer som psykologer i ett psykosocialt team. Samtliga behandlare i teamet har som lägst en grundläggande psykoterapiutbildning i KBT. I linje med primärvårdens uppdrag att utgöra en första linjens psykiatri behandlas i teamet lindriga till medelsvåra tillstånd av ångest och depression.

Forskningspersoner rekryteras från patienter som är 18 år och äldre och som söker läkare på vårdcentralen för symtom på psykisk ohälsa eller med oförklarade somatiska symtom där utredning inte leder till diagnos. Patienter som läkare på vårdcentralen bedömer att psykisk ohälsa är primär problematik remitteras till det psykosociala teamet för bedömning, diagnostik och behandling. Behandlare genomför klinisk intervju och strukturerad diagnostik med Mini-Internationell Neuropsykiatrisk Intervju (MINI; Sheehan et al., 1998). MINI är en validerad, strukturerad intervju för diagnostik och differentialdiagnostik av framförallt ångest- och depressionstillstånd (SBU, 2012).

Patienter som bedöms ha GAD som primärdiagnos och kontrollerats mot inklusions- och exklusionskriterier ges information om studien och erbjuds deltagande i den.

*Inklusionskriterier*

1. Ålder minst 18 år.
2. Behärska svenska språket utan tolkstöd.

*Exklusionskriterier*

1. Pågående användning av narkotika eller icke-förskrivna receptbelagda läkemedel (minst en månad rena prover och sex veckor för cannabis).
2. Svårare psykiatrisk störning, som bipolär sjukdom eller psykos, svårare depressivitet, suicidrisk samt diagnostiserad kognitiv funktionsnedsättning.
3. Behandling med psykofarmaka som initieras eller förändras mindre än sex veckor före behandling eller under behandling. Pågående stabil medicinering under behandling accepteras.
4. Annan samtidig psykologisk behandling.

Patienter som önskar delta i studien får muntlig och skriftlig information och ger sitt skriftliga samtycke till det. Därefter får de besvara ett batteri med instrument (självskattningsenkäter) och randomiseras sedan till behandling med MCT eller IUT. Randomisering sker av en till projektet oberoende person. En forskningssjuksköterska samlar in patienternas svar på instrument, meddelar dem vilken behandling som de har lottats till samt bokar in dem på behandlare. Båda behandlingarna är manualbaserade med ett specificerat sessionsinnehåll och kan omfatta upp till 12 sessioner. Behandlingen genomförs individuellt. En annan behandlare än den som gjorde den diagnostiska bedömningen behandlar patienten. Om patienten inte längre bedöms uppfylla kriterierna för överdriven och okontrollerbar oro kan behandlingen avslutas före den tolfte sessionen. Ljudinspelningar görs av terapisessioner under pilotstudien med patienternas medgivande enbart för att bedöma kompetens hos terapeuter och följsamhet till manualer.

*Behandlingsarmar:*

1. IUT enligt Dugas och Robichauds behandlingsmanual (2006), upp till 12 sessioner

2. MCT enligt Wells behandlingsmanual (2008), upp till 12 sessioner

*Undersökningsvariabler*

Behandlare som deltar i studien besvarar en enkät med frågor om utbildning, klinisk erfarenhet, etc. (bilaga 5 a). Patienter som deltar i studien besvarar en enkät med frågor om utbildning, hur länge de haft problem med oro, tidigare genomförd psykologisk behandling, etc. (bilaga 5 b). Psykiatrisk diagnos hos patienter bedöms av behandlare med MINI. Kompetens i MCT mäts med Metacognitive Therapy Competency Scale (Nordahl & Wells, 2009), kompetens i IUT mäts med Cognitive Therapy Scale-Revised (Blackburn, et al., 2001). Följsamhet till behandling mäts med checklista för sessionsinnehåll (se exempel i bilaga 12). Patienters upplevelser av att ha deltagit i behandlingen mäts med ett utvärderingsformulär (bilaga 13). Effektutvärderingen sker med följande fyra självskattningsinstrument:

1) patologisk oro mäts med Penn State Worry Questionnaire (PSWQ; Meyer, et al., 1990),

2) depressiva symtom med Patient Health Questionnaire 9 (PHQ-9; Kroenke, et al., 2001),

3) funktionsnivå med WHODAS 2.0 (Socialstyrelsen, 2015) och

4) livstillfredsställelse/livskvalitet med Satisfaction with Life Scale (SWLS; Diener et al., 1985).

Totalantal frågor i de fyra instrumenten är 46, vilket beräknas ta c:a 15 minuter att fylla i.

Administreringstidpunkt för instrumenten:

| Instrument | Antal frågor | Före behandling | Mellanmätning efter session 6 | Vid behandlings-avslut | Uppföljning efter 6 mån |
| --- | --- | --- | --- | --- | --- |
| PSWQ | 16 | X | X | X | X |
| PHQ-9 | 10 | X | X | X | X |
| WHODAS 2.0 | 15 | X |  | X | X |
| SWLS | 5 | X |  | X | X |
| Antal frågor | 46 | 46 | 26 | 46 | 46 |

*Tidsplan*

Totalt planeras 50 patienter ingå i pilotstudien. Datainsamlingen beräknas pågå mellan april 2018 och oktober 2019.

*Databearbetning*

Resultat för frågeställningarna 1- 4 baseras på data från de av patienterna ifyllda självskattningsinstrumenten, bakgrundsenkäterna, utvärderingsenkäten, journaldata avseende följsamhet till behandling samt behandlarens diagnostiska bedömning och kommer att beskrivas deskriptivt. Likaså data från behandlarnas bakgrundenkät redovisas deskriptivt för att kunna beskriva de två behandlargrupperna. Ljudinspelningarna som görs av terapisessioner under pilotstudien används enbart för att bedöma kompetens hos terapeuter och följsamhet till manualer

Utifrån befintligt underlag och utifrån förutsättningarna planerar vi att göra en preliminär utvärdering av effekten av MCT och IUT med statistisk signifikansprövning samt beräkning av effektstorlek och andelen patienter som är kliniskt signifikant förbättrade.

*Tidigare erfarenheter*

Både IUT och MCT är validerade och potenta psykologiska behandlingsmetoder vid GAD som båda är använda inom klinisk praxis och har använts av flera av forskarna i forskargruppen. Flera av forskarna i forskargruppen har sedan tidigare mångårig erfarenhet av studier om KBT för patienter med depression och ångestsyndrom i psykiatrisk öppenvård, inklusive rekrytering, mätning och behandling.

*Tillgång till relevant säkerhet/personal*

Pilotstudien genomförs på Liljeholmens vårdcentral inom ramen för det kliniska vardagsarbetet och patienterna rekryteras bland vårdcentralens patienter. För patienterna kommer omhändertagandet inte skilja sig ifrån det de hade fått utanför studien, med undantag av att de ombeds fylla i självskattningsinstrumenten i större utsträckning än det vanligen sker samt att behandlingssessionerna spelas in. Behandlarna är anställd personal på Liljeholmens vårdcentral samt kliniskt aktiva psykologer anställda på Kompetenscentrum för psykoterapi. Behandlarna får inledningsvis regelbunden handledning i respektive metod av erfarna kliniker och handledare. En forskningssjuksköterska kommer att engageras på deltid för att följa patienterna genom studien, se till att de besvarar självskattningsinstrumenten och bokar in besök hos behandlarna.

**Etiska överväganden**

Att få en ångestdiagnos kan upplevas stigmatiserande, särskilt för de patienter som dessförinnan inte hade kunskap om att det kunde finnas en psykologisk förklaring till somatiska besvär. En eventuell negativ initial reaktion uppvägs dock av möjligheten till korrekt diagnos och därmed tillgång till adekvat behandling. Patienterna fördelas slumpmässigt till olika behandlingar och vet inte i förväg vilken typ av behandling de kommer att få. Detta begränsar deras självbestämmande, vilket annars är vägledande i klinisk behandling. Studiens frågeställningar kräver dock denna design. De två behandlingarna är dessutom båda varianter av KBT och tidigare forskning har visat att de är potenta vid behandling av ångestproblematik. Projektet syftar till att undersöka genomförbarheten i rekrytering, mätning och behandling av patienter med GAD i primärvården, kunskap som planeras utgöra underlag för en fullskalig randomiserad kontrollerad studie vars syfte är att undersöka vilken behandling (MCT eller IUT) som är mest effektiv. Patienters deltagande i studien är frivilligt och de kan när som helst avbryta utan att deras möjligheter till god vård förändras. Patienter som tackar nej till att delta eller avbryter sitt deltagande erbjuds sedvanlig behandling. Patienter uppmanas att under bedömning och behandling rapportera eventuella oönskade händelser som uppstår inom ramen för studien till Liljeholmens vårdcentrals verksamhetschef för dokumentation och åtgärd. Vi bedömer risken att obehöriga personer ska komma åt patientdata som så gott som obefintlig i projektet. Samtyckesblanketter förvaras i låsta utrymmen på Liljeholmens vårdcentral och endast verksamhetschef, behöriga forskare och forskningssjuksköterskan har tillgång till dem. Övriga patientdata som samlas in avidentifieras och endast verksamhetschef, huvudansvarig forskare och forskningssjuksköterskan har tillgång till kodnyckeln.

**Betydelse**

Psykisk ohälsa är vanlig men inte alltid uppmärksammad bland primärvårdspatienter och särskilt GAD är ofta underdiagnostiserat. Det är därför viktigt att förbättra identifiering och handläggning av tillståndet. En systematisk diagnostik av GAD i primärvården kan ge en tidigare och korrekt diagnos, vilket är förutsättningen för adekvat behandling. Det är generellt en brist på studier av psykologisk behandling av psykisk ohälsa i primärvård och det gäller även av patienter med GAD. Även om KBT är den psykologiska behandling som är mest effektiv vid GAD saknas det kunskap om vilken variant av KBT som är den mer effektiva. Projektet förväntas ge kunskap om dels en förbättrad identifiering och handläggning av patienter med GAD i primärvård, dels bidra till kunskapsbasen om effektiviteten av olika varianter av KBT. Om MCT visar sig vara minst lika effektiv som IUT innebär det att arsenalen av psykologiska behandlingar vid GAD i primärvården bör breddas. Eftersom studien genomförs i reguljär primärvård förväntas resultaten från studien kunna generaliseras till primärvården som helhet, förutsatt att de replikeras i en mer fullskalig randomiserad kontrollerad studie.

**Referenser**

**American Psychiatric Association**.(2013). MINI-D IV. Diagnostiska kriterier enligt DSM-IV. Pilgrim Press: Danderyd.

**Blackburn**, I.-M., James, I. A., Milne, D. L., Baker, C., Standart, S., Garland, A. & Reichelt, F. K. (2001). The Revised Cognitive Therapy Scale (CTS-R): Psychometric properties. *Behavioural and Cognitive Psychotherapy, 29*, 431–446.

**Diener**, E., Emmons, R. A., Larsen, R. J., & Griffin, S. (1985). The satisfaction with life scale. *Journal of Personality Assessment, 49*(1), 71–75.

**Dugas M**. ,& Robichaud, M. (2006) *Cognitive-Behavioral Treatment for Generalized Anxiety Disorder, From Science to Practice*. Routledge

**Kroenke**, K., Spitzer, R. L., & Williams, J. B. (2001). The PHQ-9: validity of a brief depression severity measure. *Journal of General Internal Medicine, 16*(9), 606-613.

**Meyer**, T. J., Miller, M. L., Metzger, R. L., & Borkovec, T. D. (1990). Development and validation of the Penn State Worry Questionnaire. *Behaviour Research and Therapy, 28*(6), 487-495.

**Nordahl**, H. M., & Wells, A. (2009). *Metacognitive Therapy Competency Scale*. Metacognitive Therapy Institute.

**Normann, N**., van Emmerik, A. A., & Morina, N. (2014). The efficacy of metacognitive therapy for anxiety and depression: a meta-analytic review. [Meta-Analysis]. *Depression and anxiety, 31*(5), 402-411.

**Roy-Byrne**, P. P., & Wagner, A. (2004). Primary care perspectives on generalized anxiety disorder. [Review]. *The Journal of clinical psychiatry, 65 Suppl 13*, 20-26.

**Sadeghi,** R., Mokhber, N., Mahmoudi, L. Z., Asgharipour, N., & Seyfi, H. (2015). A systematic review and meta-analysis on controlled treatment trials of metacognitive therapy for anxiety disorders. [Review]. *Journal of research in medical sciences : the official journal of Isfahan University of Medical Sciences, 20*(9), 901-909.

**SBU**. Diagnostik och uppföljning av förstämningssyndrom. En systematisk litteraturöversikt. Stockholm: Statens beredning för medicinsk utvärdering (SBU); 2012. SBU-rapport nr 212. ISBN 978-91-85413-52-2.

**Sheehan**, D. V., Lecrubier, Y., Sheehan, K. H., Amorim, P., Janavs, J., Weiller, E., et al. (1998). The Mini-International Neuropsychiatric Interview (M.I.N.I.): the development and validation of a structured diagnostic psychiatric interview for DSM-IV and ICD-10. [Review]. *The Journal of clinical psychiatry, 59 Suppl 20*, 22-33;quiz 34-57.

**Socialstyrelsen.** Mätning av hälsa och funktionshinder 2015. Manual till WHO:s formulär för bedömning av funktionshinder, WHO Disability Assessment Schedule. WHODAS 2.0 Artikelnr 2015-5-1.

**Socialstyrelsen.** Nationella riktlinjer för vård vid depressions- och ångestsyndrom 2017 − stöd för styrning och ledning. Stockholm: Socialstyrelsen; 2017. Artikelnr 2017-12-4.

**van der Heiden**, C., Muris, P., & van der Molen, H. T. (2012). Randomized controlled trial on the effectiveness of metacognitive therapy and intolerance-for-uncertainty therapy for generalized anxiety disorder. *Behavior Research and Therapy, 50*, 100-109.

**Wells**, A. (2008). *Metacognitive Therapy for Anxiety and Depression*. New York: Guilford Press.

**Wittchen**, H. U. (2002). Generalized anxiety disorder: prevalence, burden, and cost to society. *Depression and anxiety, 16*(4), 162-171.
