# Supplementary material for: Intolerance-of-uncertainty therapy versus metacognitive therapy for generalized anxiety disorder in primary health care: A randomized controlled pilot trial
Source: PLoS One. 2023 Jun 14;18(6):e0287171. doi: 10.1371/journal.pone.0287171 (PMC10266649; doi:10.1371/journal.pone.0287171)
Supplement: S3 File — (DOCX) [file pone.0287171.s004.docx]

**Translated Ethical application Stockholm 2018-03-05. Dnr: 2018/505-31/1**

**Project Title:**

Comparison of the effectiveness Intolerance-of-uncertainty therapy versus metacognitive therapy for generalized anxiety disorder in primary health care: a randomized controlled feasibility pilot trial

**Responsible researchers and resources:**

**PI:** Benjamin Bohman, Med dr, psychologist, Center for Psychotherapy, Center for Psychiatry Research, Department of Clinical Neuroscience, Karolinska Institutet, & Stockholm Health Care Services, Region Stockholm, Liljeholmstorget 7, 117 63 Stockholm, e-post: benjamin.bohman@ki.se, tel: 072-244 49 00.

Sandra af Winklerfelt-Hammarberg, GP, PhD-student KI, Head of Liljeholmen primary care center, Liljeholmstorget 7. plan 4. 117 94 Stockholm, e-post: sandra.af.winklerfelt.hammarberg@ki.se,

Eva Toth-Pal, GP, Med dr, Academic coordinator at Liljeholmen primary care center, Liljeholmstorget 7. plan 4. 117 94 Stockholm, e-post: evatothpal@gmail.com

Tobias Lundgren, Fil dr, psychologist, psychotherapist. Head of Center for Psychotherapy, Center for Psychiatry Research, Department of Clinical Neuroscience, Karolinska Institutet, & Stockholm Health Care Services, Region Stockholm, Liljeholmstorget 7, 117 63 Stockholm, e-post: tobias.lundgren@ki.se

Markus Jansson-Fröjmark, Associate professor, psychologist. Center for Psychotherapy, Center for Psychiatry Research, Department of Clinical Neuroscience, Karolinska Institutet, & Stockholm Health Care Services, Region Stockholm, Liljeholmstorget 7, 117 63 Stockholm, e-post: markus.jansson-frojmark @ki.se

All participating researchers are employed within the Region of Stockholm.

Operations manager of Liljeholmen Primary Care Center, Sandra af Winklerfelt Hammarberg is responsible for the safety of the research participants. Certificate is attached (appendix 9).

Benjamin Bohman, Tobias Lundgren and Markus Jansson-Fröjmark, have many years of experience in studies on CBT for patients with depression and anxiety disorders in psychiatric outpatient care, including recruitment, measurement and treatment.

The project has received funding (SEK 250,000) from the Region of Stockholm (SLL) 2018.

**Background**

Mental health problems are increasing in the population in Sweden and constitutes significant suffering for the individual as well as high costs for healthcare and society in general (Socialstyrelsen, 2017). Generalized anxiety disorder (GAD) is a commonly occurring condition in primary care and estimates show that up to 25% of primary care patients seeking mental illness meet the criteria. GAD is characterized by excessive and uncontrollable anxiety about a number of different events or activities. The condition is often associated with increased irritability, restlessness, sleep disturbance, concentration difficulties and muscular tension with secondary pain. The condition has a chronic course and often leads to long-term suffering and functional impairment. Unfortunately, diagnostics and treatment are not optimal today. Research shows that GAD is underdiagnosed in primary care, one reason may be that secondary, diffuse bodily symptoms are often the primary reason for patients seeking care. This leads to unnecessary somatic investigations and treatment attempts.

Cognitive behavioral therapy (CBT) is the psychological treatment that has been shown to be most effective in GAD. Metacognitive therapy (MCT) is a variant of CBT and is based on theories of metacognition (Wells, 2008). It has research support as effective in several psychiatric disorders, including GAD. Another variant of CBT that has been developed specifically for GAD and that research has shown to be an effective method is intolerance-of-uncertainty therapy (IUT) (Dugas & Robichaud, 2006). IUT is applied to a much higher degree than MCT in both primary care and psychiatry. At present, it is unclear which of these variants of CBT is most effective in GAD. Only one study to date has compared the effect of MCT and IUT in GAD and that was within the psychiatric outpatient psychiatry in the Netherlands (van der Heiden, 2012). The results of this study indicate that both MCT and IUT are effective treatments for GAD, but that MCT produced better results than IUT both at treatment termination and at follow-up six months later. There are no studies carried out in Sweden, nor in primary care. The need for more research that compares these treatments for a condition as common and at the same time disabling in primary care as GAD is therefore great.

**Aims and research questions**

The aim of the study is to investigate in a pilot format the conditions for conducting a larger randomized and controlled study comparing the effect of MCT and IUT in GAD in primary care. In the pilot study, the feasibility is tested by investigating the recruitment possibilities of patients, the implementation of the measurement procedure, drop-outs from the treatments and the patients' adherence to treatment (completed sessions and homework assignments). Information about the patients' experience of undergoing the treatments is also collected through a questionnaire. In addition to this, a preliminary evaluation of the effect of MCT and IUT is carried out with statistical significance testing and calculation of effect size and the proportion of patients who are clinically significantly improved. The purpose of the study is to learn lessons about patients with GAD in primary care and thereby optimize the conditions for a full-scale randomized and controlled study. The study's questions are:

1. What does the recruitment base look like among patients with GAD in primary care and is it large enough for a full-scale randomized controlled trial?

2. To what extent do patients answer instruments for measuring symptoms, function and quality of life?

3. To what extent do patients complete the treatments and how large is the dropout rate?

4. What are the patients' experiences of undergoing these psychological treatments in a primary care setting?

5. What is the effectiveness of the two treatments and is it different between them?

**Methods**

Liljeholmen primary health care center has extensive psychotherapy services with both counselors and psychologists in a psychosocial team. All therapists in the team have at least basic psychotherapy training in CBT. In line with the mission of primary care to constitute first-line psychiatry, the team treats mild to moderate states of anxiety and depression. Patients where the doctor at the health center assesses that mental illness is the primary problem are referred to the psychosocial team for assessment, diagnostics and treatment. Therapists conduct clinical interviews and structured diagnostics. Patients who are judged to have GAD as a primary diagnosis are given information about the study and are offered participation in it. Patients who wish to participate in the study receive oral and written information and give their written consent to it. They then have to answer a battery of instruments (self-assessment questionnaires) and are then randomized to treatment with MCT or IUT. Both treatments are manual based with a specified session content and can include up to 12 sessions. A different therapist than the one who made the diagnostic assessment treats the patient. If the patient is no longer judged to meet the criteria for excessive and uncontrollable anxiety, treatment can be terminated before the twelfth session. Before the start of the study, three therapists are given training in MCT and three therapists in IUT. The therapists are also given training in diagnostics before the study starts. During treatment, therapists are given guidance in each method on a regular basis. Once during the treatment period, after completion of treatment and again six months after completion of treatment, the patients answer the same battery of instruments as during the pre-treatment.

Outcome variables

Therapists participating in the study answer a questionnaire with questions about education, clinical experience, etc. (Appendix 5 a). Patients participating in the study answer a questionnaire with questions about education, how long they have had problems with anxiety, previously completed psychological treatment, etc. (Appendix 5 b). Psychiatric diagnosis in patients is assessed by clinicians using the MINI International Neuropsychiatric Interview (MINI; for reference to this and other instruments mentioned below, see Appendix 2). Before treatment, after 5 completed sessions, after completion of treatment and at follow-up 6 months later, patients answer the following instruments, all of which are well established and validated: pathological worry is measured with the Penn State Worry Questionnaire (PSWQ), depressive symptoms with the Patient Health Questionnaire (PHQ- 9). In addition, the following instruments are answered before and after treatment and at follow-up: measurement of functional level with WHODAS 2.0 and life satisfaction/quality of life with Satisfaction with Life Scale (SWLS).

Time schedule

Data collection is expected to take place between April 2018 and October 2019.

**Possible knowledge gains**

Mental illness is common but not always noticed among primary care patients and GAD in particular is often underdiagnosed. It is therefore important to improve identification and handling of the condition. A systematic diagnosis of GAD in primary care can provide an earlier and correct diagnosis, which is the prerequisite for adequate treatment. There is generally a lack of studies of psychological treatment of mental illness in primary care and this also applies to patients with GAD. Although CBT is the psychological treatment that is most effective in GAD, there is a lack of knowledge about which variant of CBT is the more effective. The project is expected to provide knowledge about improved identification and handling of patients with GAD in primary care and contribute to the knowledge base about the effectiveness of different variants of CBT. If MCT proves to be at least as effective as IUT, it means that the arsenal of psychological treatments for GAD in primary care should be broadened. As the study is carried out in regular primary care, it is expected that the results from the study can be generalized to primary care as a whole.

1. What does the recruitment base look like among patients with GAD in primary care and is it large enough for a full-scale randomized controlled trial?

2. To what extent do patients answer instruments for measuring symptoms, function and quality of life?

3. To what extent do patients complete the treatments (sessions completed and homework assignments) and how large is the dropout rate?

4. What are the patients' experiences of undergoing these psychological treatments in a primary care setting?

5. What is the effectivness of the two treatments and does it differ between them in terms of symptoms of pathological anxiety, general anxiety and depression, and level of functioning and life satisfaction?

**Procedure**

Patients where doctors at Liljeholmen primary health carecenter assess to have mental illness as a primary problem are referred to the psychosocial team for assessment, diagnostics and treatment. Therapists conduct clinical interviews and structured diagnostics with MINI. Patients who are judged to have GAD as a primary diagnosis and checked against inclusion and exclusion criteria are given oral and written information about the study and are offered participation in it. Patients who wish to participate in the study are contacted by a research nurse and booked in for a visit. During the visit, the patient signs a consent form and then answers a battery of instruments, then the patient is randomized to MCT or IUT. Randomization is done by a person independent of the project. The research nurse collects the patients' responses to instruments, notifies them of the treatment they have been allocated to and books them in with a therapist (other than the one who carried out the assessment). After finishing treatment and again six months after finishing treatment, the patients are contacted by the research nurse to answer the same battery of instruments as before treatment.

Data sources are the patient's self-report and patient record as well as the therapist's diagnostic assessment. All data is quantifiable. Self-report includes psychiatric symptoms, level of functioning and quality of life as well as responses to a questionnaire with information on age, education, etc. Therapists also answer such a questionnaire. The research nurse is available when the patient answers instruments and can answer any questions and monitors that all questions are answered. With the MINI, the presence of psychiatric diagnoses can be determined according to a structured and validated decision procedure. The self-report instruments have scoring instructions. Questionnaires with information about therapists and patients are developed within the framework of the project and can be found in appendices 5a and 5b. Patients answer instruments before treatment, once during the treatment period and after treatment and six months after the end of treatment, estimated time consumption per occasion is 15 minutes. The data collection differs from clinical routine at the health care center to the extent that patients do not usually answer instruments in connection with treatment to this extent. In addition to the diagnostic interview and self-assessment instrument, information is collected about the patient's age, sex, and possible drug treatment and sick leave from the patient's medical record. All data collected is de-identified; personal data and the research subjects' answers are connected with a unique code. All collected data is stored in locked areas at Liljeholmen primary health care center.

Patients included in the study are assigned a unique code. A code key is established with which the patient's identifying information and code can be linked together. The code key is only available in paper format at Liljeholmen primary health care center in a locked area. Only authorized researchers, the health care center's operations manager and the research nurse have access to the code key. The research nurse is responsible for patients answering instruments before, during and after treatment and six months after the end of treatment, the nurse is also responsible for patients being randomized to treatment and booked in to therapists. Code key and collected data are stored separately in locked areas at Liljeholmen primary health care center. Only the study's researcher, the operations manager and the research nurse will have access to collected data (in de-identified condition). Karolinska Institutet's procedures for storage time and whether material will be de-identified or destroyed will be followed.

Audio recordings are made of therapy sessions during the pilot study with patients' consent solely to assess therapist competence and adherence to manuals.

**Recruitment of participants**

Participants are recruited from among patients who are 18 years of age or older and who seek treatment at Liljeholmen primary health care center. The patients are first assessed by doctors according to usual routine. Patients undergo investigation with somatic status and laboratory tests if necessary to rule out somatic causes. If the doctor then assesses that mental illness is the primary problem, the patient is referred to the health care center's psychosocial team for assessment and diagnostics. Therapists in the psychosocial team assess the patient with a clinical interview and MINI. If GAD is assessed as the primary diagnosis, the patient is given oral and written information about the study and is asked to participate, provided the patient meets the inclusion criteria but not the exclusion criteria (see below). If a diagnosis other than GAD is assessed as primary, the existing treatment routines are followed. If GAD is assessed as the primary diagnosis, the following inclusion and exclusion criteria must be checked:

**Inclusion criteria**

1. Age at least 18 years.

2. Master the Swedish language without interpreter support.

**Exclusion criteria**

1. Ongoing use of narcotics or non-prescription prescription drugs (at least one month clean samples and six weeks for cannabis).

2. More severe psychiatric disorder, such as bipolar disorder or psychosis, more severe depression, suicidality or diagnosed cognitive impairment.

3. Treatment with psychotropic drugs that is initiated or changed less than six weeks before treatment or during treatment. Ongoing stable medication during treatment is accepted.

4. Other concurrent psychological treatment.

Researchers have no direct relationship with the research participants. Researchers neither assess nor treat the participants. On the other hand, one of the participating researchers is also the operations manager at Liljeholmen's primary health care center where the therapists participating in the study work, which should not, however, entail a risk of impact on the patients' treatment.

The participation of patients in the project forms part of their regular healthcare. The same insurance cover applies as for routine healthcare. Participants pay no patient fee for the study visits including assessment and treatment sessions, but other than that receive no economic compensation for participation.

**Informed consent**

The research nurse asks for consent during a visit when patients who want to participate in the study must answer instruments before treatment and be randomized to MCT or IUT and booked in to a therapist. Signed consents are collected and stored in locked areas at Liljeholmen primary health care center. Operations manager, participating researchers and the research nurse have access to them. For written research person information, see appendix 4.

**Ethical considerations**

Receiving an anxiety diagnosis can be experienced as stigmatizing, especially for those patients who previously had no knowledge that there could be a psychological explanation for somatic complaints. However, a possible negative initial reaction is outweighed by the possibility of a correct diagnosis and thus access to adequate treatment. Patients are randomly allocated to different treatments and do not know in advance which type of treatment they will receive. This limits their self-determination, which is otherwise indicative in clinical treatment. However, the study's questions require this design. In addition, the two treatments are both variants of CBT and previous research has shown them to be potent in the treatment of anxiety problems. The project aims to investigate the feasibility of recruiting, measuring and treating patients with GAD in primary care, knowledge that is planned to form the basis for a full-scale randomized controlled trial whose purpose is to investigate which treatment (MCT or IUT) is most effective. Patients' participation in the study is voluntary and they can cancel at any time without changing their opportunities for good care. Patients who refuse to participate or cancel their participation are offered usual treatment. During assessment and treatment, patients are asked to report any unwanted events that occur within the scope of the study to Liljeholmen primary care center's operations manager for documentation and action. We assess the risk of unauthorized persons accessing patient data as virtually non-existent in the project. Consent forms are kept in locked areas at Liljeholmen primary health care center and only the operations manager, authorized researchers and the research nurse have access to them. Other patient data that is collected is de-identified and only the operations manager, authorized researchers and the research nurse have access to the code key.

Participants in the project can benefit from undergoing an adequate assessment and a structured diagnostic procedure and are thus given the opportunity for effective treatment. GAD is a commonly occurring condition in primary care and the benefits of the project, including structured assessment and diagnostics as well as manual-based potent psychological treatment, can be expected to benefit many patients.

Access to data is guaranteed because participating researchers have control over data collection and data documentation. Participating researchers are responsible for data processing and report writing. The project is planned to result in a manuscript that is submitted for publication in a scientific journal. Results are reported at group level and no individual patients will be able to be identified in the planned publication. Patient data is de-identified through the use of serial numbers that are linked to personal data through a code key that only the operations manager, participating researchers and the research nurse within the project have access to. The code key is stored in locked areas at Liljeholmen primary health care center.

**Power calculation**

As this is a pilot study, assessment of effectiveness is not of primary interest and thus a power calculation is not of decisive importance either. In the pilot study, we plan to include a total of approximately 50 patients who are recruited at Liljeholmen primary health care center with the aim of investigating the feasibility of the method by studying the recruitment possibilities of patients, the implementation of the interest measurement procedure, dropouts from the treatments and the patients' adherence to treatment (completed sessions and homework assignments). Based on existing data and based on the conditions, however, we also plan to make a preliminary evaluation of the effectiveness of MCT and IUT with statistical significance testing and calculation of effect size and the proportion of patients who are clinically significantly improved.

**References**

Dugas M. ,& Robichaud, M. (2006) Cognitive-Behavioral Treatment for Generalized Anxiety Disorder, From Science to Practice. Routledge

Socialstyrelsen. Nationella riktlinjer för vård vid depressions- och ångestsyndrom 2017 − stöd för styrning och ledning. Stockholm: Socialstyrelsen; 2017. Artikelnr 2017-12-4.

van der Heiden, C., Muris, P., & van der Molen, H. T. (2012). Randomized controlled trial on the effectiveness of metacognitive therapy and intolerance-for-uncertainty therapy for generalized anxiety disorder. Behavior Research and Therapy, 50, 100-109.

Wells, A. (2008). Metacognitive Therapy for Anxiety and Depression. New York: Guilford Press
