# Supplementary material for: Intolerance-of-uncertainty therapy versus metacognitive therapy for generalized anxiety disorder in primary health care: A randomized controlled pilot trial
Source: PLoS One. 2023 Jun 14;18(6):e0287171. doi: 10.1371/journal.pone.0287171 (PMC10266649; doi:10.1371/journal.pone.0287171)
Supplement: Attachment — Submitted filename: Response to reviewers 230323.docx [file pone.0287171.s005.docx]

Dear Associate professor Walid Kamal Abdelbasset and the Editorial Board,

Thank you for the opportunity to revise and resubmit the article, “Intolerance-of-uncertainty therapy versus metacognitive therapy for generalized anxiety disorder in primary health care: a randomized controlled pilot trial” (submission ID PONE-D-22-31734) to PLOS ONE.

In response to the journal requirements, we have adjusted the manuscript to meet the PLOS ONE’s style requirements, including those for file naming and figures.

Data cannot be shared publicly because of Swedish legal and ethical restrictions related to sensitive patient information. Data are available from Region Stockholm (contact via data protection officer Camilla Heise Löwgren, camilla.heise-lowgren@regionstockholm.se) for researchers who meet the criteria for access to confidential data. Therefore, we have changed the data availability statement.

According to PLOS ONE’s Editorial Policy, we have added the original and a translated version of the study protocol to the translated ethical application in the supporting information files.

Please find our point-by-point responses to the reviewers below. Changes in the manuscript are highlighted in grey.

Sincerely,

Sandra af Winklerfelt Hammarberg on behalf of all the authors

**Reviewers' comments:**

Reviewer's Responses to Questions

**Comments to the Author**

1. Is the manuscript technically sound, and do the data support the conclusions?

The manuscript must describe a technically sound piece of scientific research with data that supports the conclusions. Experiments must have been conducted rigorously, with appropriate controls, replication, and sample sizes. The conclusions must be drawn appropriately based on the data presented.

Reviewer #1: Partly

Reviewer #2: Yes

2. Has the statistical analysis been performed appropriately and rigorously?

Reviewer #1: Yes

Reviewer #2: Yes

3. Have the authors made all data underlying the findings in their manuscript fully available?

The [PLOS Data policy](https://eur01.safelinks.protection.outlook.com/?url=http%3A%2F%2Fwww.plosone.org%2Fstatic%2Fpolicies.action%23sharing&data=05%7C01%7Csandra.af.winklerfelt.hammarberg%40ki.se%7Cc835e3e7b9634a08b54108db08063438%7Cbff7eef1cf4b4f32be3da1dda043c05d%7C0%7C0%7C638112595098122680%7CUnknown%7CTWFpbGZsb3d8eyJWIjoiMC4wLjAwMDAiLCJQIjoiV2luMzIiLCJBTiI6Ik1haWwiLCJXVCI6Mn0%3D%7C3000%7C%7C%7C&sdata=b1unUE7q4WrRtJXShFnjRTIikHYSDT6uhm7O2gVzI1k%3D&reserved=0) requires authors to make all data underlying the findings described in their manuscript fully available without restriction, with rare exception (please refer to the Data Availability Statement in the manuscript PDF file). The data should be provided as part of the manuscript or its supporting information, or deposited to a public repository. For example, in addition to summary statistics, the data points behind means, medians and variance measures should be available. If there are restrictions on publicly sharing data—e.g. participant privacy or use of data from a third party—those must be specified.

Reviewer #1: Yes

Reviewer #2: Yes

**Authors Response:** Data cannot be shared publicly because of Swedish legal and ethical restrictions related to sensitive patient information. Data are available from Region Stockholm (contact via data protection officer Camilla Heise Löwgren, camilla.heise-lowgren@regionstockholm.se) for researchers who meet the criteria for access to confidential data. Therefore, we do not have the opportunity to provide files or links to these data and have changed the data availability statement.

4. Is the manuscript presented in an intelligible fashion and written in standard English?

PLOS ONE does not copyedit accepted manuscripts, so the language in submitted articles must be clear, correct, and unambiguous. Any typographical or grammatical errors should be corrected at revision, so please note any specific errors here.

Reviewer #1: Yes

Reviewer #2: Yes

5. Review Comments to the Author

Please use the space provided to explain your answers to the questions above. You may also include additional comments for the author, including concerns about dual publication, research ethics, or publication ethics. (Please upload your review as an attachment if it exceeds 20,000 characters)

**Reviewer #1:** The current paper describes a pilot RCT in comparing two interventions (IUT vs. MCT) based on CBT to treat GAD in the primary care setup. The paper is well written and follows the general guidelines of the pilot trials quite well. Some minor discrepancies were observed which is described in the review below.

**Response:** Thank you.

**Comment:** 1. Pilot RCT was not meant to evaluate either efficacy or effectiveness. Hence, correct the statement in line 162.

**Response:** A secondary aim was to evaluate preliminary effectiveness, the sentence in line 162 has been revised accordingly.

**Comment:** 2. Both interventions are delivered via trained therapists. However, it is not clear how many therapists delivered the therapy for each arm. It is also not clear if a therapist is assigned to a specific subject throughout the study. This will have implications is the statistical analysis plan based on what is actually done and the therapist-level effect may need to be evaluated in the statistical model.

**Response:** Participants were randomized to either IUT or MCT and received treatment from the same therapist throughout treatment, which was another therapist than the therapist performing the first assessment before randomization (lines 175 to 176.) However, therapists self-selected which treatment they would deliver. There were four therapists who self-selected to the IUT arm and six who self-selected to the MCT arm as described in line 186, a clarification has been added in line 187. We believe there is not sufficient power in our pilot trial to include a therapist effect in the models because of few therapists and few participants per therapist.

**Comment:** 3. In a similar context it is not clear if a therapist is used to deliver both IUT and MCT to a different subject. Mixing up therapists may or may not affect contamination between the arms.

**Response:** We agree in the importance of these aspects and thus, no mixing of therapists was allowed in the study. We have tried to clarify this further in the methods section line 187: “Therapists provided either IUT or MCT throughout the whole trial”, and in the result section lines 400 to 401: “the four IUT therapists treated eight participants each (range=4-12), and the six MCT therapists five participants each (range=2-10)”.

**Comment:** 4. CONSORT diagram must also include all the data collection points including the primary endpoint and primary outcome(s).

**Response:** We have revised Fig 1, the CONSORT flow diagram and added the collection points including primary (feasibility) and secondary preliminary effectiveness outcomes. In keeping with the principle of intention-to-treat, data from all participants were used in the multilevel models. Missing data were estimated using the maximum likelihood estimation (lines 339 to 342)

**Comment:** 5. It is reported that a multi-level model (also known as a mixed-effect model). is used. Please clearly state which is random and which one is a fixed effect (and why). Should your model also include the therapist-level effect?

**Response**: We started with a basic model with a fixed intercept, then we built models adding random intercept and slope, and finally an interaction term. Allowing intercepts to vary (i.e., random intercepts model) means that the outcome variable is at different levels prior to treatment (i.e., the symptom level varies across participants); this is similar to including pre-treatment symptom level as a covariate in analysis of covariance. Similarly, allowing slopes to vary means that the change trajectory may vary across participants. Finally, to investigate whether treatment condition moderated the effect, an interaction term of time (fixed effect; treatment period) and treatment condition (fixed effect; IUT or MCT) was added to the model with random intercept and slope. This has been clarified on lines 320-327**.** We believe there is not sufficient power in our pilot trial to include a therapist effect in the models because of few therapists and few participants per therapist.

**Comment:** 6. Since the pilot study is not an efficacy and/or effectiveness-seeking study and not powered to detect any effect all the references of statistical significance, such as but not limited to p-value should be removed. The result of statistical details (line 351+) should be rewritten in light of the pilot/feasibility trial.

**Response:** Because a secondary aim of the study was to explore preliminary effects, we would prefer to retain p-values and confidence intervals. Although the study was not powered to detect statistically significant effects, we believe that the interpretation of effects and effect sizes is facilitated by providing measures of statistical significance (i.e., p-values and confidence intervals). It is more the rule than the exception that pilot studies report statistical significance of effects.

**Comment:** 7. The authors may also give some specific details or key takeaways of the change/adaption that they recommend for conducting the larger powered trial from the lesson learned from the pilot/feasibility trial.

**Response:** We agree in the importance of these aspects, which we discuss in separate parts of the discussion section. Most important in the future RCT is including more primary health care centers (line 586) and to do a power calculation for effectiveness measures (lines 618 to 619). Therapists’ competence could be tested before they are included in treatment, and therapists could be randomized rather than selecting protocol themselves (lines 615 to 618). Moreover, improvements of the inter-rater reliability in the assessments of therapist competence and adherence are needed. We intend to assess more sessions by at least two assessors for each measure as described in lines 566 to 570. In this feasibility study, we did not assess potential mediators of effect, which could be of interest to include in the future RCT (line 589 to 590). In lines 622 to 628 we discuss assessing further important outcomes for patients including somatic symptoms and use of care in a future study, and in lines 631 to 632 we underscore the importance of monitoring pharmaceutical treatment.

**Reviewer #2:** I would like to thank authors for their effort to conduct such a research work, however, I have some comments to be raised as follows:
**Comment:**- the authors did not mention how they calculate their sample size as even for the pilot studies there are rules for sample size calculation(1). please add it in details .

1- Whitehead AL, Julious SA, Cooper CL, Campbell MJ. Estimating the sample size for a pilot randomised trial to minimise the overall trial sample size for the external pilot and main trial for a continuous outcome variable. Stat Methods Med Res. 2016 Jun;25(3):1057-73. doi: 10.1177/0962280215588241. Epub 2015 Jun 19. PMID: 26092476; PMCID: PMC4876429.

**Response:** Thank you. The primary aim was to test feasibility, i.e. to test whether something can be done rather than to investigate the preliminary effects which typically can be the aim of a pilot [1]. As there was uncertainty about the feasibility of recruiting participants to an RCT in this setting, we set out to investigate the flow of recruitment as a feasibility measure. However, we set a goal of recruiting at least 50 participants to enable us to assess feasibility and preliminary effectiveness. In their paper, Whitehead and colleagues state that the “focus of this paper will be deriving pilot trial sample sizes based on a primary aim of the pilot being to estimate the standard deviation to be used for the main trial sample size calculation” (page 1058). This was not the primary aim of our pilot study; rather, it was to investigate the feasibility of a full-scale effectiveness trial in terms of participant recruitment and retention, willingness to receive psychological treatment, and therapists’ competence in and adherence to treatment protocols (lines 140 to 144). Thus, it seems that the paper of Whitehead and colleagues is not relevant in this context. Basing sample size on a primary aim of feasibility similar to ours is an acceptable procedure according to the CONSORT extension to randomized pilot and feasibility trials (Eldridge, et al., 2016). Moreover, there is controversy over the appropriateness of using data from pilot studies to inform sample size calculation in full-scale effectiveness trials ([2] e.g., Leon, Davis, & Kraemer, 2011).

- Statistical analysis section:
-------------------------------

**Comment:** -did the authors investigate the normality of data before proceeding in the inferential statistics used ??? as it was not mentioned in the original manuscript?!!

**Response:** Thank you for pointing that out. Yes, we investigated the normality of all outcome variables pre-treatment. A clarification has been added in line 313.

**Comment:** authors did not mention the statistical strategies for managing missing data?? please add it to manuscript and explain how it had been managed.

**Response:** In the manuscript, it is stated that “missing data were estimated using maximum likelihood estimation” (line 340). This estimation is automatic and integrated in the multilevel modeling procedure in SPSS. Additions have been made to clarify (lines 340 to 341). On lines 379 to 382, results are presented concerning missing values.

- Intervention:
-----------------
More details are needed even if the study followed previous published protocols ( references 30&35) to avoid distraction of the readers

**Response**: The contents of IUT and MCT are described in Table 1. The text in lines 97, 99 and 100 of the theory and methods of IUT has been clarified. An example has been added in lines 100-101 to clarify methods of MCT.

6. PLOS authors have the option to publish the peer review history of their article ([what does this mean?](https://eur01.safelinks.protection.outlook.com/?url=https%3A%2F%2Fjournals.plos.org%2Fplosone%2Fs%2Feditorial-and-peer-review-process%23loc-peer-review-history&data=05%7C01%7Csandra.af.winklerfelt.hammarberg%40ki.se%7Cc835e3e7b9634a08b54108db08063438%7Cbff7eef1cf4b4f32be3da1dda043c05d%7C0%7C0%7C638112595098122680%7CUnknown%7CTWFpbGZsb3d8eyJWIjoiMC4wLjAwMDAiLCJQIjoiV2luMzIiLCJBTiI6Ik1haWwiLCJXVCI6Mn0%3D%7C3000%7C%7C%7C&sdata=FKd44oeMQLKssThXE21VwsDAIOMD%2FMIcJyd4gF%2Fg0AQ%3D&reserved=0)). If published, this will include your full peer review and any attached files.

If you choose “no”, your identity will remain anonymous but your review may still be made public.
**Do you want your identity to be public for this peer review?** For information about this choice, including consent withdrawal, please see our [Privacy Policy](https://eur01.safelinks.protection.outlook.com/?url=https%3A%2F%2Fwww.plos.org%2Fprivacy-policy&data=05%7C01%7Csandra.af.winklerfelt.hammarberg%40ki.se%7Cc835e3e7b9634a08b54108db08063438%7Cbff7eef1cf4b4f32be3da1dda043c05d%7C0%7C0%7C638112595098122680%7CUnknown%7CTWFpbGZsb3d8eyJWIjoiMC4wLjAwMDAiLCJQIjoiV2luMzIiLCJBTiI6Ik1haWwiLCJXVCI6Mn0%3D%7C3000%7C%7C%7C&sdata=3HS4io8JvatgGydt0UBh5rWkdLMDUlY80DUmfW%2B71dQ%3D&reserved=0).

Reviewer #1: No

Reviewer #2: No

**References:**

 1.	Eldridge SM, Lancaster GA, Campbell MJ, Thabane L, Hopewell S, Coleman CL, et al. Defining Feasibility and Pilot Studies in Preparation for Randomised Controlled Trials: Development of a Conceptual Framework. PloS one. 2016;11(3):e0150205.

2.	Leon AC, Davis LL, Kraemer HC. The role and interpretation of pilot studies in clinical research. Journal of psychiatric research. 2011;45(5):626-9.
