# Supplementary material for: Intolerance-of-uncertainty therapy versus metacognitive therapy for generalized anxiety disorder in primary health care: A randomized controlled pilot trial
Source: PLoS One. 2023 Jun 14;18(6):e0287171. doi: 10.1371/journal.pone.0287171 (PMC10266649; doi:10.1371/journal.pone.0287171)
Supplement: Attachment — Submitted filename: Response to reviewers 230525.docx [file pone.0287171.s006.docx]

Dear Associate professor Ethan Moitra and the Editorial Board,

Thank you for the opportunity to revise and resubmit the article, “Intolerance-of-uncertainty therapy versus metacognitive therapy for generalized anxiety disorder in primary health care: a randomized controlled pilot trial” (submission ID PONE-D-22-31734R1) to PLOS ONE.

In response to the journal requirements, we have revised the manuscript to meet the comments of the reviewers. Please find our point-by-point responses to the reviewers below. Changes in the revised manuscript are highlighted in grey.

Sincerely,

Sandra af Winklerfelt Hammarberg on behalf of all the authors

**Reviewers' comments:**

Reviewer's Responses to Questions

**Comments to the Author**

1. If the authors have adequately addressed your comments raised in a previous round of review and you feel that this manuscript is now acceptable for publication, you may indicate that here to bypass the “Comments to the Author” section, enter your conflict of interest statement in the “Confidential to Editor” section, and submit your "Accept" recommendation.

**Reviewer #2:** All comments have been addressed

2. Is the manuscript technically sound, and do the data support the conclusions?

The manuscript must describe a technically sound piece of scientific research with data that supports the conclusions. Experiments must have been conducted rigorously, with appropriate controls, replication, and sample sizes. The conclusions must be drawn appropriately based on the data presented.

**Reviewer #2:** Partly

3. Has the statistical analysis been performed appropriately and rigorously?

**Reviewer #2:** Yes

4. Have the authors made all data underlying the findings in their manuscript fully available?

The [PLOS Data policy](http://www.plosone.org/static/policies.action#sharing) requires authors to make all data underlying the findings described in their manuscript fully available without restriction, with rare exception (please refer to the Data Availability Statement in the manuscript PDF file). The data should be provided as part of the manuscript or its supporting information, or deposited to a public repository. For example, in addition to summary statistics, the data points behind means, medians and variance measures should be available. If there are restrictions on publicly sharing data—e.g. participant privacy or use of data from a third party—those must be specified.

**Reviewer #2:** Yes

5. Is the manuscript presented in an intelligible fashion and written in standard English?

PLOS ONE does not copyedit accepted manuscripts, so the language in submitted articles must be clear, correct, and unambiguous. Any typographical or grammatical errors should be corrected at revision, so please note any specific errors here.

**Reviewer #2:** Yes

6. Review Comments to the Author

Please use the space provided to explain your answers to the questions above. You may also include additional comments for the author, including concerns about dual publication, research ethics, or publication ethics.

**Reviewer #2:** thank you for your responding to the raised comments, however, there are some comments to be raised:
Regarding the sample size calculation; being the primary aim of the study is to investigate the feasibility, this does not give you an excuse for lacking sample size calculation. If the study was following CONSORT guidelines as described in manuscript and clarifies in supporting files (S1), so, the sample size calculation should be addressed. Please address this as a major limitation in the limitation section as this will affect generalizability of the study.

**Response:** Thank you for your valuable comments. We have revised the limitation section (lines 619 to 623 on page 28) to clarify this major limitation. The section now reads: “Finally, no sample size calculation was conducted, as the primary aim of the present study was to investigate feasibility. The mostly large effect sizes nevertheless suggest that the preliminary results regarding treatment effects are reliable. However, the lack of a sample size calculation constitutes a major limitation that may affect generalizability of the results.”
